# High lactose whey cheese consumption and risk of colorectal cancer - The Norwegian Women and Cancer Study

**DOI:** 10.1038/s41598-018-36445-6

**Published:** 2019-01-22

**Authors:** Runa Borgund Barnung, Mie Jareid, Marko Lukic, Sunday Oluwafemi Oyeyemi, Jan Håkon Rudolfsen, Evgeniya Sovershaeva, Guri Skeie

**Affiliations:** Department of Community Medicine, Faculty of Health Sciences, UiT – The Arctic University of Norway, Tromsø, Norway

## Abstract

The incidence of colorectal cancer (CRC) has increased among Norwegian women, and is among the highest in the world. In order to understand this increase, country specific dietary exposures have been investigated. The aim of this study was to quantify the association between consumption of brown cheese, a common bread topping in Norway, and colorectal, colon, and rectal cancer in the prospective Norwegian Women and Cancer (NOWAC) Study. Data on brown cheese consumption and adjustment factors was available for 82 527 women. During a mean of 14.6 years of follow-up (1.2 million person-years), there were 1360 cases of colorectal cancer (907 colon; 453 rectal). Multivariable Cox proportional hazards models were used to calculate hazard ratios (HR) with 95% confidence intervals (CI) for colorectal, colon, and rectal cancer sites. We modelled restricted cubic splines with 4 knots, to assess a possible non-linear relationship between brown cheese intake and the investigated cancer sites. In the age-adjusted model, consumption of more than 2 slices (>16 grams) of brown cheese per day was associated with 13% reduced risk of colon cancer (95% CI 4%-21%) compared to women who did not consume brown cheese. The multivariable-adjusted model, however, showed no association between brown cheese consumption and the risk of colorectal, colon, or rectal cancer (colorectal: HR = 0.93, 95% CI 0.76–1.13, *p*-trend 0.37; colon: HR = 0.83, 95% CI 0.65–1.06; *p*-trend = 0.10; rectal: HR = 1.16, 95% CI 0.84–1.1.61, *p*-trend = 0.41). In this large, prospective cohort study of women, consumption of brown cheese was suggestively protective against colon cancer. However, adjustment attenuated the inverse risk association. Brown cheese consumption was not associated with rectal cancer, or colorectal cancer overall.

## Introduction

Norway has seen a steep rise in colorectal cancer (CRC) incidence in the past six decades. Among Norwegian women, the incidence has risen from 9.9 per 100 000 in 1955–59, to 52.5 per 100 000 in 2011–2015^1^. The rise in CRC incidence has been steeper in Norway than in the neighboring countries^2^. The cumulative risk of CRC until age 75 is now 4.1%^3^. It is thought that dietary and lifestyle risk factors for CRC, such as obesity, inactivity, and refined foods, induce a systemic inflammatory state that potentiates the carcinogenesis^4^.

One potential cause of the high Norwegian CRC incidence could be population specific exposures. In this paper we set out to examine the association between one country-specific exposure, brown cheese, and CRC. Brown cheese – a bread-topping specific to Norway – has, to the best of our knowledge, not been studied as a risk factor for cancer.

Brown cheese is made from whey, the remnants after production of white cheese. While other whey cheeses, such as ricotta, are made by separating the whey protein from the liquid, brown cheese is unique in that it is made by evaporating the liquid. Brown cheese thus contains all the constituents of whey, and is very high in lactose (35–47%)^5^. Lactose is a disaccharide of D-glucose and D-galactose. D-galactose may induce chronic inflammation in doses corresponding to 4–6 slices of brown cheese^6,7^. Dairy products are widely consumed in Norway, and are, predominantly, negatively associated with CRC^8^. Though few studies have investigated the effect of lactose on risk of CRC^9,10^, these studies reported no association between high and low lactose intake and CRC risk.

We sought to assess whether brown cheese could be a contributing factor to the high incidence of CRC in Norwegian women, by using the Norwegian Women and Cancer (NOWAC) Study, a nationally representative prospective cohort of women with comprehensive dietary information.

## Methods

### Study cohort

The NOWAC cohort was initiated in 1991 and consists of a random sample of 172,000 Norwegian women, aged 30–70 years at baseline, who completed a questionnaire regarding their lifestyle, diet, and health status (overall response rate: 52.7%). Detailed information on the NOWAC Study is available elsewhere^11^. For this study, we used information from women who completed baseline food frequency questionnaires (FFQ) in 1996–1997, 1998, 2003, and 2004. The NOWAC Study was approved by the Regional Committee for Medical Research Ethics, and the Norwegian Data Inspectorate. The participants received written information on the study, and the mailing of a completed questionnaire was regarded as informed consent. All research was performed in accordance with relevant guidelines/regulations.

Our initial sample consisted of 101 321 women. We excluded women that had missing information on brown cheese consumption (N = 1423), those with prevalent cancer other than non-melanoma skin cancer at baseline, and those who emigrated or died before the start of follow-up (N = 4603). Those with total energy intake above 15 000 kJ or below 2500 kJ per day (N = 608), and with missing on covariates (N = 12 160) were also excluded. The study sample included 82 527 women.

### Assessment of brown cheese consumption

The questionnaire asked “On how many slices of bread do you use brown cheese per week/day?”. The participants were encouraged to include brown cheese eaten with other foods in the estimate. Brown cheese consumption was divided into 2 questions, one for full fat brown cheese and one for fat reduced brown cheese. The frequency of consumption was the same for both questions, and was divided into 6 categories: 0 per week, 1–3 per week, 4–6 per week, 1 per day, 2–3 per day, and 4 + per day.

Total brown cheese consumption was calculated as the combined consumption of full fat brown cheese and fat reduced brown cheese. Assuming 2 slices of brown cheese per slice of bread, and one slice brown cheese weighing 8 grams^7^, consumption was then recalculated into grams per day. Finally, for analytical purposes we categorized total brown cheese consumption as ‘never’, ‘up to 2 slices of brown cheese/day’, and ‘more than 2 slices/day’.

### Cancer incidence, death, and emigration

We obtained information on cancer incidence, death, and emigration in the cohort through linkage to the Norwegian Cancer Registry, the Cause of Death Registry, and the Norwegian Central Population Register, respectively. The unique 11-digit personal number assigned to every legal resident in Norway was used for the linkage, but concealed from researchers. We included invasive cancers of the colon (C18) and rectum (C19-C20) as defined by the 10^th^ Revision of the International Statistical Classification of Diseases, Injuries and Causes of Death.

### Statistical analysis

We calculated person-years from start of follow-up until diagnosis of any incident cancer other than non-melanoma skin cancer, until death, emigration, or the end of the study period (31^st^ December 2015), whichever occurred first.

Cox proportional hazards regression models were used to calculate hazard ratios (HRs) with 95% confidence intervals (CIs) for the association between brown cheese consumption and the risk for developing colorectal, colon, or rectal cancer. Those who did not report consuming brown cheese were used as reference group. We used attained age as the underlying time scale. In order to take into account potential differences during the long follow-up time (i.e. long period of baseline data collection, differences in questionnaires, and participant age), all models were stratified by questionnaire subcohorts. The proportional hazards assumption was checked by visual inspection of the log-minus-log survival plot, which did not indicate violation of the assumption.

Analyses for each cancer outcome were adjusted for known risk factors^8,12,13^ in the preliminary analyses. In the full model, we adjusted for the following covariates: body mass index (BMI, ≤18.49, 18.5–24.9, 25–29.9, and ≥30 kg/m2), intake of processed meat (tertiles, g/day), intake of red meat (≤10, 10.01–20, >20 g/day), height (continuous, cm), smoking status (never, former, current), intake of fiber (≤20, >20 g/day), alcohol consumption (0, 0.1–3.99, 4–9.99, ≥10 g/day), hormone replacement therapy use (never, former, current), total energy intake (tertiles, kJ/day), physical activity level (1–4, 5–6, 7–10), diabetes mellitus (yes/no), intake of hard white cheese (quartiles, g/day), estimated calcium intake (tertiles, mg/day), intake of milk (tertiles, g/day), intake of fish (quartiles, g/day), and intake of whole grain bread (tertiles, g/day). If the removal of a covariate changed the regression coefficient by at least 10% in any of the brown cheese consumption categories, it was retained in the multivariable models.

To test for linear trend, a median value was calculated for each category of the ordinal brown cheese consumption variable, which was then modeled as a continuous variable in the analysis. To assess a possible non-linear relationship between the main exposure and the study outcomes, we modeled restricted cubic splines with four knots, with its locations based on Harrell’s recommended percentiles of the brown cheese consumption^14^. We then used a Wald-type test to assess if the coefficients of the second and third spline were equal to zero. We also tested for interaction effect between brown cheese and all the variables in the final model. This did not change the results or improve the fit of the model.

To check for possible reverse causality, we excluded cancer cases of interest that were diagnosed during the first two years of follow-up, and repeated the analyses. As part of sensitivity analyses, we repeated the analyses with all covariates included in the model, regardless of whether they previously fulfilled the 10% criterion. Additionally, we ran the model under the assumption that a slice of brown cheese equates to 10 g or 6 g (as opposed to 8 g), and evaluated energy intake, bread, milk, and alcohol consumption, and calcium level as continuous variables. In order to control for cancer cases occurring at advanced age, we also estimated the model with censoring of individuals when reaching 75 years of age (n censored = 11 239). Finally, we estimated the model using either months or days as timescale. We evaluated the sensitivity of the model based on the significance of the HRs and log-likelihood (as basis for chi square test of fit) for all sensitivity testing.

Analyses were performed using Stata v14.0 (StataCorp, College Station, Texas) and R v3.4.0 (www-r-project.org).

## Results

During the mean 14.6 years of follow-up and 1.2 million person-years, 1360 cases of colorectal cancer occurred, of which 907 (66.7%) were colon cancer and 453 (33.3%) were rectal cancer cases. The majority (59%) of the women in the study cohort consumed up to 2 slices (≤16 grams) of brown cheese per day. The consumption of milk and whole grain bread was higher among women who reported eating more than 2 slices of brown cheese per day, compared to non-consumers and those who reported eating up to 2 slices per day. Women who consumed more than 2 slices had higher total energy intake (8176 kJ/day) compared to non-consumers (6587 kJ/day) and those who consumed up to 2 slices per day (7072 kJ/day) (Table 1).

**Table 1:** Selected characteristics of the study sample by brown cheese consumption, the Norwegian Women and Cancer Study, 1996–2015 (N = 82 527).

Characteristics	Brown cheese consumption		
	Never	Up to 2 slices/day	More than 2 slices/day
Participants at baseline, N (%)	24 689 (29.9)	48 689 (59.0)	9149 (11.1)
Colorectal cancer, N (%)	398 (1.61)	814 (1.67)	148 (1.62)
Colon cancer, N (%)	270 (1.09)	546 (1.12)	91 (0.99)
Rectal cancer, N (%)	128 (0.52)	268 (0.55)	57 (0.62)
Age at baseline (y), mean (SD)	51.8 (6.2)	51.6 (6.4)	51.9 (6.7)
Age at study exit (y), mean (SD)	66.0 (6.6)	66.3 (6.9)	67.2 (7.2)
Brown cheese intake (g/day), mean (SD)	0	8.6 (4.7)	38.6 (10.1)
**Smoking status at baseline, N (%)**			
Never	7601 (30.8)	18 911 (38.8)	3984 (43.5)
Former	8267 (33.5)	16 222 (33.3)	2992 (32.7)
Current	8821 (35.7)	13 556 (27.8)	2173 (23.7)
**Physical activity level, N (%)**			
1–4	7489 (30.3)	12 415 (25.5)	2089 (22.8)
5–6	10 228 (41.4)	21 441 (44.0)	3849 (42.1)
7–10	6972 (28.2)	14 833 (30.5)	3211 (35.1)
Alcohol consumption (g/day), mean (SD)	4.2 (5.0)	3.6 (4.4)	2.8 (3.8)
**Use of hormone replacement therapy at baseline, N (%)**			
Never	15 532 (62.9)	32 257 (66.2)	6216 (67.9)
Former	3344 (13.5)	5704 (11.7)	979 (10.7)
Current	5813 (23.5)	10 728 (22.0)	1954 (21.4)
Total energy intake (kJ/day), mean (SD)	6586.7 (1772.9)	7072.1 (1794.1)	8176.1 (1912.5)
**Quartile of milk consumption, N (%)**			
1 (low)	12 680 (51.4)	20 623 (42.4)	3742 (40.9)
2 (middle)	6603 (26.7)	14 178 (29.1)	2449 (26.8)
3 (high)	5406 (21.9)	13 888 (28.5)	2958 (32.3)
**Whole grain bread, N (%)**			
1 (low)	17 676 (71.6)	32 947 (67.7)	4359 (47.6)
2 (middle)	6013 (24.3)	13 848 (28.4)	3852 (42.1)
3 (high)	1000 (4.1)	1894 (3.9)	938 (10.2)
Calcium, mean (SD)	695.6 (300.1)	740.8 (288.7)	955.3 (345.2)
Height, mean (SD)	166.1 (5.7)	166.2 (5.6)	166.4 (5.6)

SD: standard deviation.

In the age-adjusted model, consumption of more than 2 slices of brown cheese per day was associated with 13% reduced risk of colon cancer (95% CI 4%-21%). There was also a statistically significant trend (p = 0.03) of decreasing colon cancer incidence with higher intake of brown cheese. However, the association was no longer statistically significant after multivariable adjustment (HR = 0.83, 95% CI 0.64–1.06; p-trend across categories = 0.10). Brown cheese consumption was not associated with risk of colorectal or rectal cancer, neither in the age-adjusted or in the multivariable adjusted model. (Table 2).

**Table 2:** Hazard ratios (HRs) with 95% confidence intervals (CI) of colorectal, colon, and rectal cancer according to brown cheese consumption in the Norwegian Women and Cancer Study, 1996–2015 (N = 82 857).

Brown cheese consumption	Colorectal cancer n = 1360		Colon cancer n = 907		Rectal cancer n = 453	
	Age-adjusted	Multivariable^1^	Age-adjusted	Multivariable^1^	Age-adjusted	Multivariable^1^
	HR 95% CI	HR 95% CI	HR 95% CI	HR 95% CI	HR 95% CI	HR 95% CI
Never	1.00	1.00	1.00	1.00	1.00	1.00
Up to 2 slices/day	0.99 (0.88–1.11)	1.02 (0.91–1.16)	0.97 (0.84–1.22)	1.01 (0.87–1.17)	1.02 (0.83–1.27)	1.06 (0.86–1.31)
More than 2 slices/day	0.86 (0.71–1.04)	0.93 (0.76–1.13)	0.87 (0.79–0.96)	0.83 (0.64–1.06)	1.07 (0.78–1.47)	1,16 (0.84–1.61)
*p*_trend_	0.11	0.37	0.03	0.10	0.68	0.41

^1^Adjusted for smoking status (cat.), physical activity level (cat.), alcohol consumption (g/day) (cat.), use of hormone replacement therapy (cat.), milk consumption (g/day) (tertiles), total energy intake (kJ/day) (tertiles), whole grain bread consumption (g/day) (tertiles), estimated calcium intake (mg/day) (tertiles), and height (centimeters) (cont).

Cat.: categorical; cont.: continuous.

**Figure 1:**
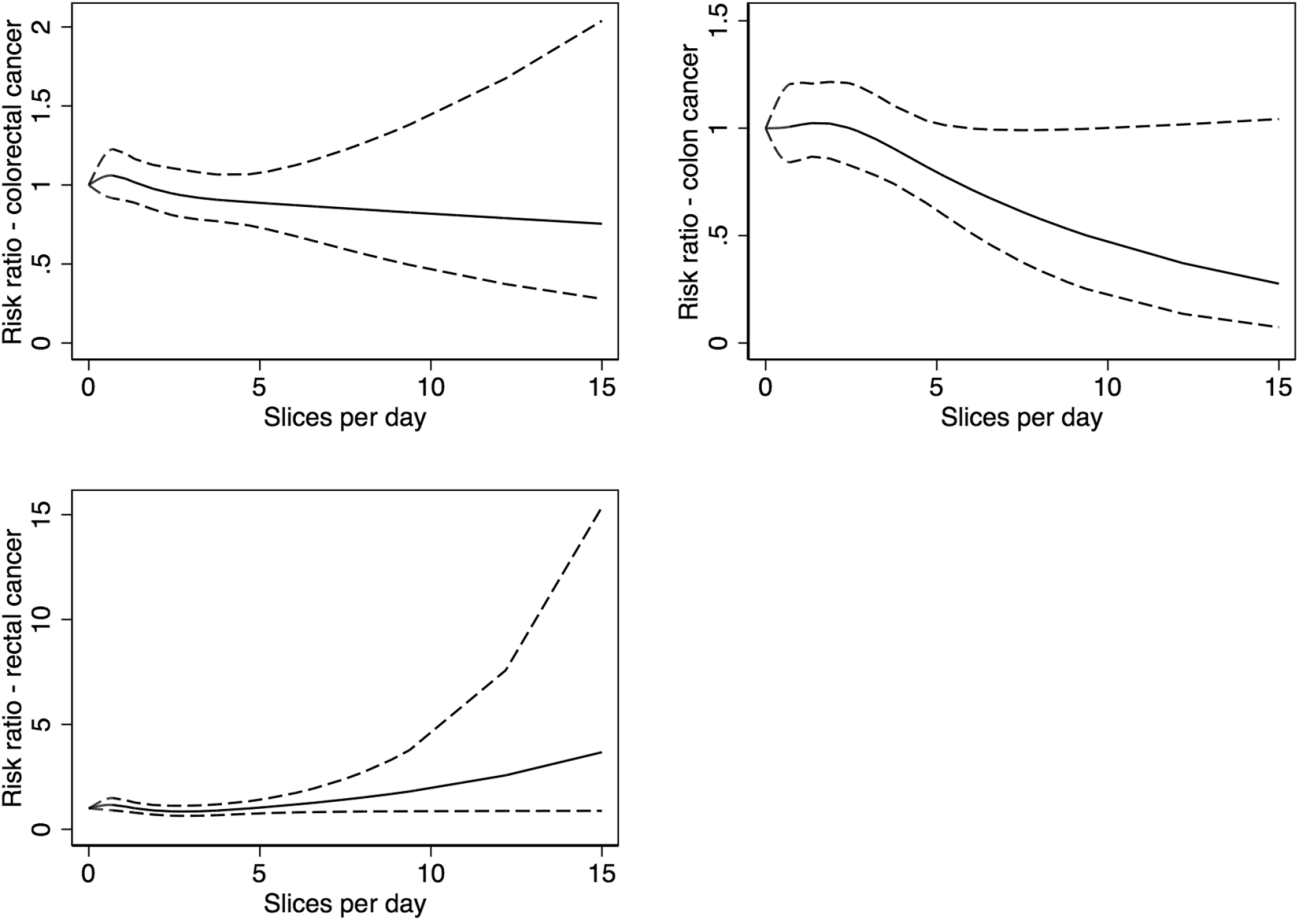
Spline regression models for brown cheese consumption in relation to colorectal, colon, and rectal cancer. (Solid lines - HR, dashed lines - 95% CI).

We did not observe any departure from linearity between brown cheese intake and the risk of colorectal, colon, or rectal cancer (p = 0.57, p = 0.41, p = 0.07 respectively) (Fig. 1).

The relationships remained the same when those who had a cancer of interest diagnosed during the first two years of the follow-up were excluded from the analyses (data not shown). The observed estimates did not change after we conducted the analyses in which we additionally included the covariates that did not change the coefficients of the brown cheese consumption categories more than 10% (data not shown). None of the measures taken during the sensitivity analysis affected the results.

## Discussion

In this nationally representative prospective cohort of Norwegian women, consumption of more than 2 slices of brown cheese per day was associated with 13% reduced risk of colon cancer in the age-adjusted model, while multivariable adjusted risk estimates showed no association between consumption of brown cheese and the risk of colorectal, colon, or rectal cancer.

Several cohort studies have looked at carbohydrate intake, often together with glycemic index (GI), and CRC. Many found no association for neither carbohydrates nor GI^15,16^. This was also what was reported by the Continuous Update Project (CUP)^17^. Higginbotham *et al*.^18^ found that carbohydrates, but not GI, might increase the risk of CRC. However, Strayer *et al*.^19^ found a reduction in colorectal cancer risk for diets high in carbohydrates and in GI. These authors also studied GI from dairy and non-dairy separately, and found a stronger inverse association with the GI from dairy sources, while the GI from non-dairy had a weaker and non-significant inverse association. They suggest that this inverse association is likely due to the high calcium content found in most dairy products. Brown cheese contains 500 mg/100 g calcium^20^. Dietary calcium is inversely associated with CRC, and it is suggested as one of the main mechanisms for dairy having a probable protective effect on CRC^8^.

In our study, participants with the highest consumption of brown cheese also had a higher intake of milk and calcium. This could potentially explain the protective effect of brown cheese on colon cancer in the age-adjusted model.

CUP’s dose-response meta-analysis^17^ found a 13% decreased risk of CRC per 400 g of dairy products per day. This is in concurrence with another meta-analysis on dairy intake and CRC risk^21^. The WCRF therefore states that dairy probably decreases CRC risk^17^. However, in one meta-analysis, no association was found between consumption of non-fermented milk, fermented milk, or solid cheese and colon or rectal cancer in women^22^. Two cohort studies reported significant inverse associations between dairy products and CRC^23,24^, whereas others found no relation between dairy products and overall CRC risk in their cohort^25,26^. One study found that intake of dairy was inversely associated with rectal cancer irrespective of total calcium intake, while total calcium was inversely associated with colon cancer irrespective of dairy consumption^27^. From 1972–2001, brown cheese was fortified with iron (10 mg/100 g) as ferrous sulfate^20^. We found no studies that investigated the association between consumption of iron-fortified products and risk of CRC.

The heaviest consumers of brown cheese in our study were also slightly more physically active, and consumed more fish and wholegrain bread. These are markers of a healthy lifestyle, although high consumers of brown cheese also smoked more. However, this could suggest brown cheese as a marker of a more traditional lifestyle, in which other factors contribute to lower colon cancer risk.

The strengths of this study include its prospective design, large sample size, and representativeness of the Norwegian female population^28^. Registry-based ascertainment of cancer cases ensured near complete follow-up^29^. We used self-reported dietary data for assessment of brown cheese intake. In a 24-h dietary recall study conducted to validate the FFQ used in the NOWAC Study, the median calibration coefficient for foods was 0.57, and Spearman’s r for cheese was 0.42^30^. The reliability of self-reported height and weight was found to be acceptable in a test-retest assessment^31^.

Brown cheese consumption was self-reported. Thus, there is bound to be some misclassification in dietary exposure, which is most likely non-differential. We used dietary information collected at baseline only, and participants may have changed their dietary habits. The consumption of brown cheese in Norway has steadily decreased^32^, and this could also be the case for the study participants, leading to an overestimation of the exposure. Even though we checked for confounding by important CRC risk factors, and performed rigorous adjustment, residual confounding cannot be excluded. When we conducted analyses in which we additionally adjusted for the covariates that did not change the coefficient for brown cheese more than 10%, however, the estimates did not change for any of the outcomes.

### Conclusion

In this large, prospective cohort study of Norwegian women, consumption of brown cheese was suggestively protective against colon cancer. Adjustment attenuated the inverse risk association, and brown cheese consumption was not associated with rectal cancer, or colorectal cancer overall. Despite the unique properties of this country-specific dietary component, brown cheese consumption cannot explain the high incidence of CRC in Norway.
